# Electrocardiographic imaging of His bundle, left bundle branch, epicardial, and endocardial left ventricular pacing to achieve cardiac resynchronization therapy

**DOI:** 10.1016/j.hrcr.2020.04.012

**Published:** 2020-05-04

**Authors:** Mark K. Elliott, Vishal Mehta, Baldeep Singh Sidhu, Steven Niederer, Christopher A. Rinaldi

**Affiliations:** ∗ School of Biomedical Engineering and Imaging Sciences, King’s College, London, United Kingdom; † Guy’s and St Thomas’ Hospital, London, United Kingdom

**Keywords:** Cardiac resynchronization therapy, Electrocardiographic imaging, Endocardial pacing, Heart failure, His bundle pacing, Left bundle branch pacing

## Introduction

Alternative methods of delivering cardiac resynchronization therapy (CRT), including left ventricular (LV) endocardial pacing, His bundle pacing, and left bundle branch pacing, have been developed in an effort to improve CRT. Noninvasive cardiac mapping using electrocardiographic imaging (ECGi) is a method of assessing ventricular activation and has been used in the CRT population to predict and assess response, guide LV lead placement, and optimize therapy.^1^, ^2^, ^3^, ^4^ In this report we describe the electrical effects of these different pacing modalities using ECGi.

## Case report

A 62-year-old man underwent an electrophysiology and ECGi study 21 weeks after the implantation of a St Jude Quadra Assura MP CRT-D system (St Jude Medical, Inc, St Paul, MN). This was implanted following a new diagnosis of heart failure during a hospital admission for breathlessness and syncope. Admission electrocardiogram showed sinus rhythm with left bundle branch block (LBBB) and a QRS duration of 170 ms. Transthoracic echocardiogram showed a dilated left ventricle with severely impaired systolic function (ejection fraction 25%), normal right ventricular (RV) function, and mild mitral regurgitation. He underwent a coronary angiogram, which showed unobstructed coronary arteries. He underwent cardiac magnetic resonance imaging, which confirmed the diagnosis of nonischemic cardiomyopathy with no evidence of scar on late gadolinium enhancement. The patient had a background of treated prostate cancer, hypertension, and hypercholesterolemia.

**Figure 1:**
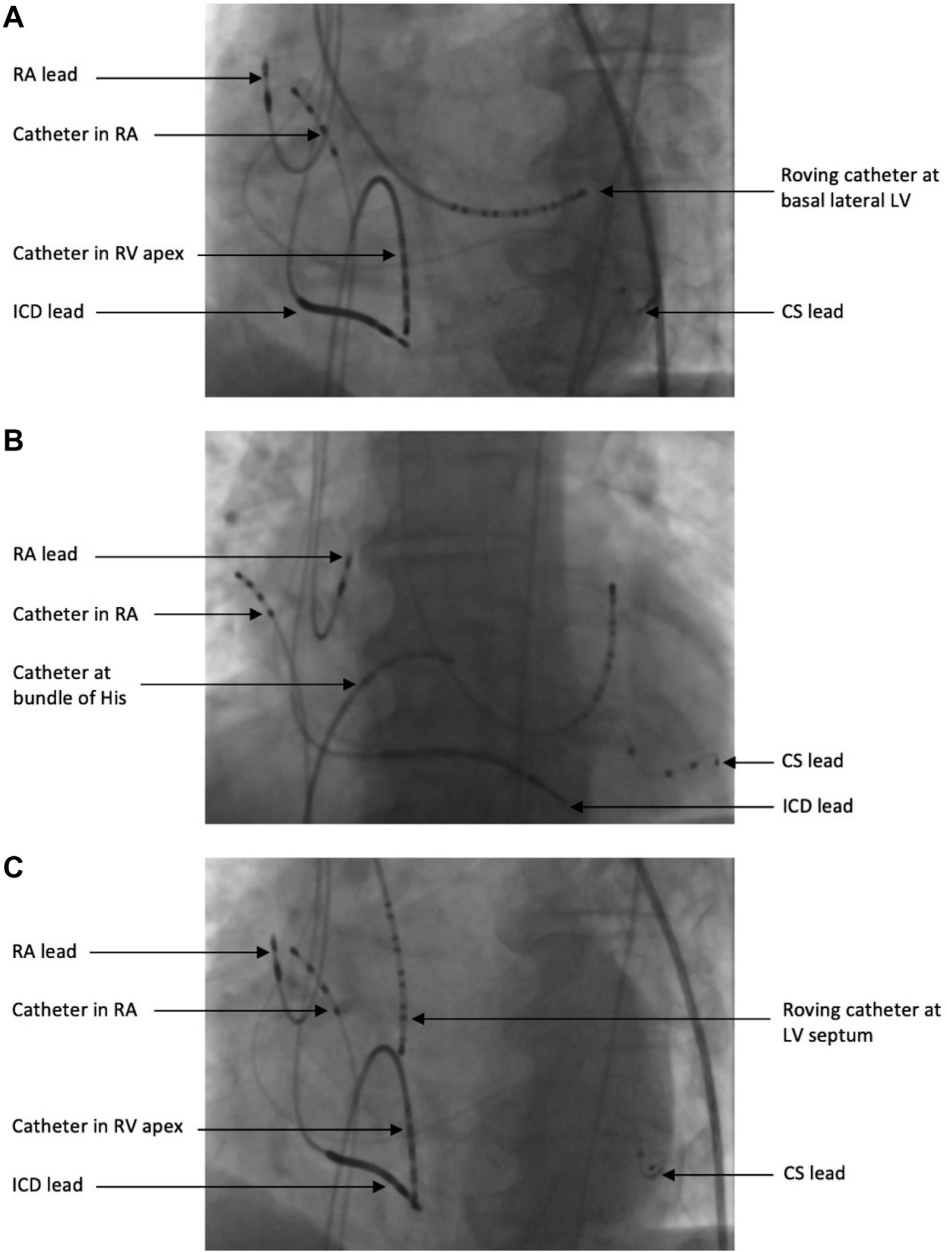
Fluoroscopy images of catheter positions. **A:** Endocardial pacing. **B:** His bundle pacing. **C:** Left bundle branch pacing. CS = coronary sinus; ICD = implantable cardioverter-defibrillator; LV = left ventricle; RA = right atrium; RV = right ventricle.

The electrophysiology and ECGi study were performed as part of a mechanistic cohort study (ClinialTrials.gov identifier NCT04322877). The patient provided written consent for the study, which was conducted in accordance with the Declaration of Helsinki and approved by the local ethics committee. The patient was fitted with a 252-electrode CardioInsight Sensor Array Vest (Medtronic, Minneapolis, MN) before the procedure and underwent a low-dose computed tomography scan to obtain electrode and cardiac positions, as previously described.^5^ Conventional CRT was delivered through the implanted CRT-D device. Temporary His bundle pacing was achieved using a high right atrial quadripolar catheter for atrial pacing and a roving decapolar catheter (6F Livewire 115 cm, St Jude Medical, Inc, St Paul, MN) to locate and pace the His bundle. Endocardial CRT was performed using the high right atrial quadripolar catheter, a roving decapolar catheter at the RV apex, and a second roving decapolar catheter (6F Livewire 115 cm, St Jude Medical, Inc) at the basal lateral wall of the left ventricle, via femoral arterial access with a retrograde aortic approach. Left bundle branch pacing was performed using the high right atrial quadripolar catheter and the roving decapolar catheter in the left ventricle by locating a left bundle signal on the septum. Fluoroscopy images of catheter positions are shown in Figure 1. All temporary pacing was performed at 10 beats per minute above intrinsic rate with a fixed output of 5 V at 0.5 ms and an atrioventricular delay of 100 ms.

Activation maps were created using the ECSYNC CardioInsight software (Medtronic, Minneapolis, MN) and the following activation parameters were calculated:

(1) Global right/left ventricular electrical synchrony (VVsync): the difference between the mean RV activation time and the mean LV activation time.
(2) Global biventricular total activation time (VVtat): a measure of the total time required for both ventricles to activate.
(3) Global left ventricular total activation time (LVtat): a measure of the total time required for the left ventricle to activate.
(4) Global left ventricular dispersion time (LVdisp): the standard deviation of LV activation times; a measure of dyssynchrony within the left ventricle.

These measures have been previously validated in patients undergoing ECGi assessment during CRT.^3^^,^^4^^,^^6^

**Figure 2:**
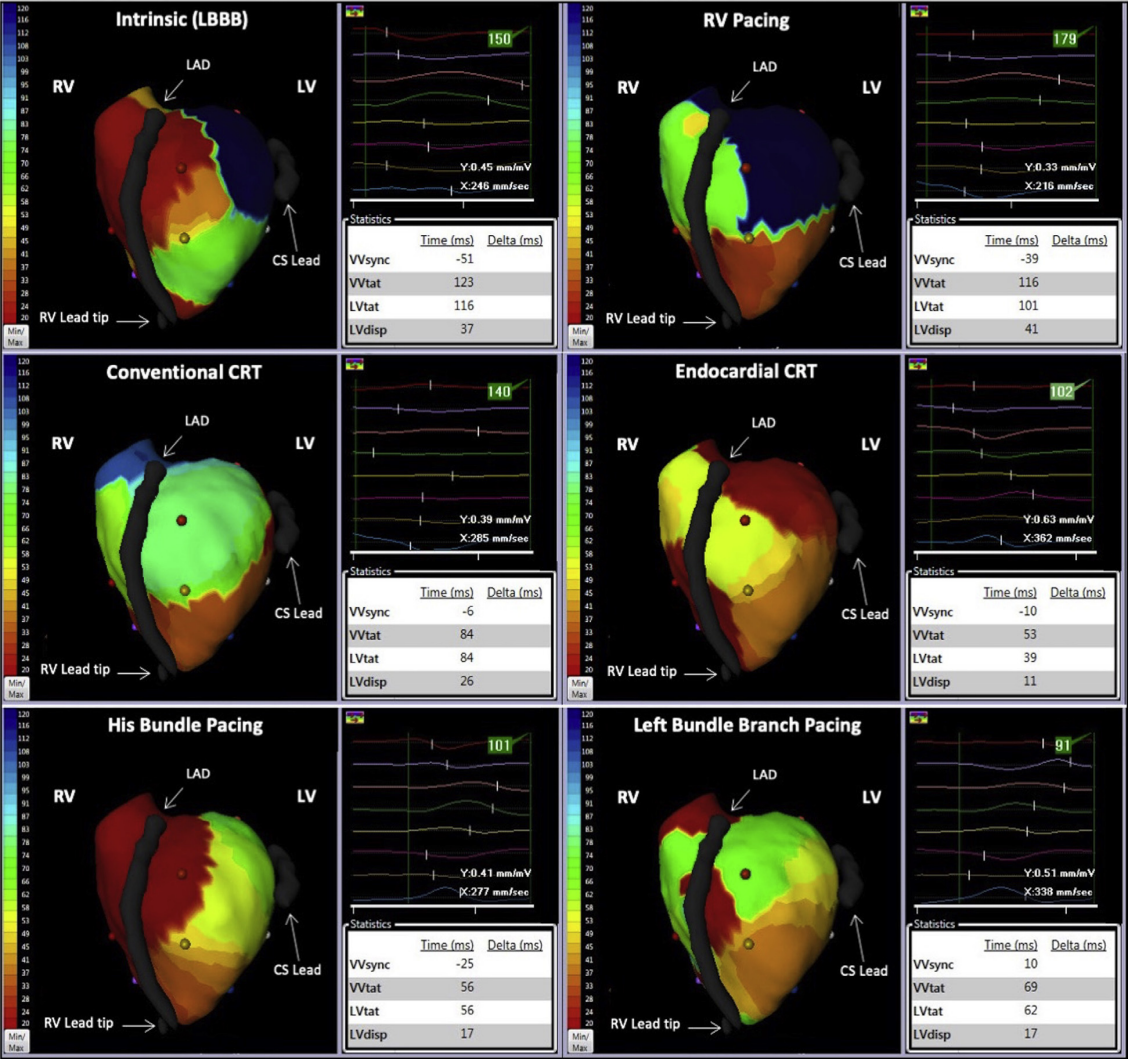
Activation maps for each pacing configuration. Early activation is shown in red and late activation is shown in blue. CRT = cardiac resynchronization therapy; CS = coronary sinus; LAD = left anterior descending artery; LBBB = left bundle branch block; LV = left ventricle; LVdisp = left ventricular dispersion time; LVtat = left ventricular total activation time; RV = right ventricle; VVsync = right/left ventricular electrical synchrony; VVtat = biventricular total activation time.

The activation maps and activation parameters are displayed in Figure 2. The maps demonstrate different activation patterns between different modes of CRT delivery. Baseline intrinsic conduction shows an LBBB pattern with slow conduction across the anterior wall and late activation of the basal lateral wall (seen in blue). A similar activation pattern is seen in RV pacing. All 4 methods of CRT resulted in reversal of LBBB. The activation maps for conventional CRT and endocardial CRT demonstrate early activation (seen in red) at the RV apex and in the mid lateral and basal lateral LV walls, respectively. In contrast, His bundle and left bundle branch pacing show early activation in the septum with rapid activation across the LV, which is consistent with recruitment of the His-Purkinje network.

As expected, intrinsic (LBBB) conduction showed dyssynchronous late activation of the left ventricle compared to the right ventricle, demonstrated by a very negative VVsync. All methods of CRT improved right/left ventricular synchrony, with VVsync having values closer to zero. In addition, all methods of CRT improved biventricular total activation (VVtat) and left ventricular activation (LVtat) times as well as dyssynchrony within the left ventricle (LVdisp). Interestingly, in this patient the improvement in VVtat, LVtat, and LVdisp was greater with endocardial CRT, His bundle pacing, and left bundle branch pacing, compared to conventional CRT.

## Discussion

This case demonstrates the feasibility and utility of ECGi to compare different methods of delivering CRT. Although there has been great interest in alternatives to conventional CRT, to our knowledge this is the first report of the electrical effects of conventional CRT, endocardial CRT, His bundle pacing, and left bundle branch pacing in the same patient. In this patient, the alternative methods of CRT delivery seemed to result in superior ventricular resynchronization compared to conventional CRT. However, the cohort of patients in whom CRT is indicated is a heterogenous group and it is unlikely that a “one-size-fits-all” method of CRT delivery will be optimal in all patients. Differences in cardiac anatomy, scar, and conduction block within the His-Purkinje system are likely to affect response to different methods of CRT delivery. An electrophysiology study with ECGi assessment may therefore be a useful endeavor to find the optimal means of CRT delivery prior to implantation.

Endocardial pacing results in more physiological activation of the myocardium and mechanistic studies have suggested an improvement in hemodynamics and activation times over conventional CRT.^7^ Our group has demonstrated that, in a group of ischemic CRT nonresponders, pacing at the same site endocardially resulted in a significantly greater acute hemodynamic response and shorter QRS duration compared to epicardially.^8^ It also allows the operator to target an optimal area of myocardium without scar and where there is late mechanical activation, without restriction by coronary venous anatomy. Clinical studies have demonstrated the feasibility and effectiveness of both wired and wireless endocardial pacing systems, though concerns remain about thromboembolic risk and procedural complications.^9^, ^10^, ^11^

Biventricular pacing results in delivery of 2 nonphysiological wavefronts, which merge to activate the myocardium. Recruitment of the His-Purkinje system to activate the ventricles is an attractive concept to achieve synchrony in the heart failure population. It has been theorized that pacing the bundle of His with sufficient output can reverse bundle branch block. Observational studies have demonstrated the feasibility and effectiveness of His bundle pacing in delivering CRT to patients with heart failure, though thresholds required for capture and bundle-branch correction are high.^12^ Arnold and colleagues^13^ directly compared His bundle pacing to conventional CRT in 23 patients with heart failure and LBBB, using ECGi and hemodynamic assessment. They found that His bundle pacing delivered better ventricular resynchronization and a better acute hemodynamic response. More recently, left bundle branch pacing has been proposed as a means to reverse LBBB at lower thresholds and has been shown to be a feasible method of CRT delivery in small observational studies.^14^^,^^15^

## Conclusion

To our knowledge, this is the first report of a direct comparison of conventional CRT, endocardial CRT, His bundle pacing, and left bundle branch pacing in the same patient. Randomized controlled trials are required to assess the superiority of these newer techniques over conventional CRT. Given the heterogeneous cohort of patients who are indicated for CRT, it is unlikely that a single technique will be optimal in every patient. An electrophysiology study with ECGi is a potential method of assessment to help tailor therapy to individual patients.

**Key Teaching Points**

• Electrocardiographic imaging (ECGi) is a noninvasive method for mapping electrical activation of the heart.
• Endocardial pacing, His bundle pacing, and left bundle branch pacing are alternative methods of delivery of cardiac resynchronization therapy (CRT) and demonstrate different activation patterns using ECGi.
• Comparison of alternative methods of CRT using an electrophysiology study and ECGi is a potential technique to tailor pacing strategy in patients who do not respond to conventional CRT.
